# Rapid quantification of insulin degludec by immunopurification combined with liquid chromatography high-resolution mass spectrometry

**DOI:** 10.1007/s00216-020-02971-4

**Published:** 2020-10-02

**Authors:** Gemma Reverter-Branchat, Michael Groessl, Christos T Nakas, Jean-Christophe Prost, Kwasi Antwi, Eric E. Niederkofler, Lia Bally

**Affiliations:** 1 Department of Diabetes, Endocrinology, Nutritional Medicine and Metabolism, Inselspital, Bern University Hospital, University of Bern, 3010 Bern, Switzerland; 2 Department of Nephrology and Hypertension, Inselspital, Bern University Hospital, University of Bern, Freiburgstrasse, 3012 Bern, Switzerland; 3 Laboratory of Biometry, School of Agriculture, University of Thessaly, 38446 Nea Ionia, Magnesia Greece; 4 University Institute of Clinical Chemistry, Inselspital, Bern University Hospital, University of Bern, 3010 Bern, Switzerland; 5 Thermo Fisher Scientific, Tempe, AZ 85280 USA

**Keywords:** Insulin degludec, Immunopurification, Mass spectrometry, Quantification

## Abstract

Insulin degludec is an ultra-long-acting insulin analogue that is increasingly being used in diabetes due to its favourable efficacy and safety profile. Thus, there is an increasing demand for a reliable and specific analytical method to quantify insulin degludec for research, pharmaceutical industry and clinical applications. We developed and validated an automated, high-throughput method for quantification of insulin degludec in human blood samples across the expected clinical range combining immunopurification with high-resolution mass spectrometry. Validation was performed according to the requirements of the US Food and Drug Administration. The method satisfyingly met the following parameters: lower limit of quantification (120 pM), linearity, accuracy (error < 5%), precision (CV < 7.7%), selectivity, carry-over, recovery (89.7–97.2%), stability and performance in the presence of other insulin analogues. The method was successfully applied to clinical samples of patients treated with insulin degludec showing a good correlation with the administered dose (*r*^2^ = 0.78). High usability of the method is supported by the small specimen volume, automated sample processing and short analysis time. In conclusion, this reliable, easy-to-use and specific mass spectrometric insulin degludec assay offers great promise to address the current unmet need for standardized insulin analytics in academic and industrial research.

**Electronic supplementary material:**

The online version of this article (10.1007/s00216-020-02971-4) contains supplementary material, which is available to authorized users.

**Figure:**
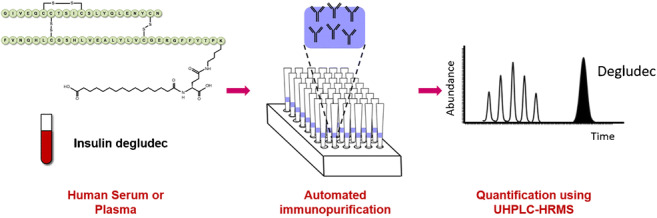
Graphical Abstract

## Introduction

Over 200 million people with diabetes are treated with insulin and this number continues to increase globally [1]. Nowadays, synthetic insulin or insulin analogues, which have modified primary sequences compared with human insulin, have become the standard of diabetes care due to their more favourable pharmacokinetic profile [2, 3]. Short-acting insulin analogues are administered to achieve peak insulin levels at mealtimes, whereas long-acting analogues (also known as basal insulins) provide a steady release of insulin over longer periods [3]. Insulin degludec (IDeg, Novo Nordisk, Bagsvaerd, Denmark) is the most recently developed long-acting insulin analogue and is a DesB30 human insulin acylated at the ε-amino group of LysB29 with hexadecandioic acid via a γ-l-glutamic acid linker [4]. This lipidation leads to self-aggregation into multi-hexamers after subcutaneous injection and association with human serum albumin in circulation [4], both contributing to the ultra-long action profile of IDeg of up to 42 h [4, 5]. Clinically, the flat and stable pharmacokinetic profile of IDeg translates into a reduced risk of hypoglycaemia and increased flexibility for administration [3, 6, 7] making it a popular agent for diabetes care.

Despite the increasing interest in this ultra-long-acting insulin formulation, the ability to reliably quantify IDeg in human blood samples currently remains a challenge. Amongst the widely available immunometric assays for insulin, only two providers show low to modest cross-reactivity with IDeg [8]. A commonly cited specific IDeg immunoassay seems to be confined to the manufacturer of the insulin formulation [9]. Regardless of their availability, all immunometric assays are unable to separate different insulin variants in a sample, suffer from interferences and have limited linear dynamic ranges [8, 10]. Thus, development of mass spectrometry–based methods that show better performance in terms of specificity, selectivity and dynamic range is of high interest [11, 12]. In this context, mass spectrometric immunoassays (MSIA) that couple immunopurification with liquid chromatography mass spectrometry (LC-MS) are currently regarded as the most promising approach [12, 13]. Although quantification of IDeg in human plasma using LC-MS has been recently demonstrated [14], a comprehensive method validation that meets the criteria set forth by the US Food and Drug Administration (FDA), essential for clinical trials, has not yet been performed. Thus, this work aimed to develop and validate a MSIA workflow for the quantification of IDeg in human blood samples that exhibits both ease of use and high performance.

## Materials and methods

### Chemicals and reagents

Formic acid, water, acetonitrile, trifluoroacetic acid (LC-MS grade) and hydrochloric acid solution (0.1 N) were from Fisher Chemical (Waltham, MA, USA). Leucine enkephalin acetate salt was from Abcam (Cambridge, UK). Bovine insulin was from Sigma-Aldrich (St. Louis, MO, USA). BupH modified Dulbecco’s PBS Packs (PBS), 10 mM PBS (pH 7.4), *n*-octyl-β-d-glucopyranoside (NOG), Nunc 2.0 mL DeepWell plates, Nunc 96-well polypropylene plates and 500 μL insulin MSIA D.A.R.T.’S (Disposable Automation Research Tips) 96-rack were from Thermo Scientific (Tempe, AZ, USA). Bovine serum albumin (BSA) (Fraction V, cold ethanol precipitated) was from Fisher BioReagents (Waltham, MA, USA). For calibrants and quality control (QC), insulin-free, charcoal-stripped, delipidized human serum was from Cone Bioproducts (Seguin, TX, USA). For selectivity assessment, serum and plasma from the following sources were tested: sodium EDTA (pooled) plasma, potassium EDTA (pooled) plasma and serum (pooled) from BioIVT (Hicksville, NY, USA); sodium heparin plasma (pooled) from ProMedDx (Norton, MA, USA); stripped human serum (non-pooled) from Biocell (Rancho Dominguez, CA, USA). All insulin formulations were purchased from the Hospital Pharmacy: IDeg and insulin aspart (IAsp) were from Novo Nordisk Pharma (Bagsvaerd, Denmark). Insulin glulisine (IGlu) and insulin lispro (ILisp) were from Sanofi-Aventis (Gentilly, France).

### Clinical samples

Fasting EDTA plasma samples were collected from 14 male adults with type 1 diabetes (age 31 ± 8 years, body mass index 25.6 ± 3.2 kg/m^2^) treated with basal IDeg. Patients provided written informed consent and sample collection was approved by the local Ethics Committee (2018-02070). All patients were at a stable IDeg regimen and injected 24 ± 9 units of IDeg (Tresiba U-100, Novo Nordisk Pharma, Bagsvaerd, Denmark) 8.7 ± 5.0 h before sample collection. Plasma was separated by centrifugation within 30 min of collection and stored at − 80 °C until analysis.

### Preparation of calibrants and quality control samples

Commercial IDeg was provided at a concentration of 600 μM and stored at − 20 °C in 20-μL aliquots. Calibrants were prepared by serial dilution in stripped serum at 120, 250, 500, 1000, 2000, 4000, 6000 and 8000 pM. QC samples were at 120 pM (LLOQ), 360 pM (QC low), 1500 pM (QC mid) and 7000 pM (QC high). Concentrations were chosen to cover the full range of expected therapeutic IDeg levels in patients with type 1 and type 2 diabetes [5]. Bovine insulin was prepared at a concentration of 697.7 μM in 0.1 N HCl to be used as an internal standard (ISTD). In total, 20-μL aliquots were created for storage at − 20 °C. For each assay, a fresh aliquot was thawed and serially diluted in 150 nM NOG in 10 mM PBS, pH = 7.4. For measurement, 250 μL of sample (calibrants, QC or patient samples) was used. In total, 250 μL of 800 pM bovine insulin was added to each sample as ISTD. For blank samples, 250 μL of 10 mM PBS was used instead of serum.

### Degludec immunoprecipitation using insulin MSIA D.A.R.T.’S

IDeg immunoprecipitation from serum samples was achieved using insulin MSIA D.A.R.T.’S mounted onto a Versette automated liquid handler (Thermo Scientific, USA) equipped with a 96 head as previously described with minor variations [15]. In contrast to the previously published method, only 250 μL instead of 500 μL is used and the following sequence was used for the automatized immunoprecipitation of IDeg from serum: MSIA D.A.R.T.’S were initially rinsed with 10 mM PBS, pH 7.4 (20 cycles, 150-μL aspiration/dispensation volume each). For immunocapture, the serum samples were aspirated using the rinsed MSIA D.A.R.T.’S (100 cycles, 250-μL aspiration/dispensation volume each). After immunocapture, tips were rinsed twice with PBS 10 mM, pH 7.4 (20 cycles, 150-μL aspiration/dispensation volume each) and twice with water (20 cycles, 150-μL aspiration/dispensation volume each) (see Electronic Supplementary Material (ESM) Table S1). Elution of the immunoprecipitated insulin was performed in 60 μL of an acidic solution (100 cycles, 50-μL aspiration/dispensation volume each) consisting of 0.4% trifluoroacetic acid and 33% acetonitrile containing 450 μg/mL leucine enkephalin and 25 mg/L BSA to help stabilize eluates post-elution. Eluates were directly placed in the autosampler for LC-MS analysis. The total procedure took 1 h and 25 min to be completed. To verify robustness, MSIA D.A.R.T.’S from three different lots (19290622, 19300529 and 19401400) were used during the validation procedure.

### Quantification of insulin by liquid chromatography high-resolution mass spectrometry

Liquid chromatography was performed using a Thermo Vanquish UHPLC system with a ProSwift RP-4H LC column 1 × 50 mm (both Thermo Scientific, USA) maintained at 70 °C. Solvent A was 0.2% formic acid in water and solvent B was 0.2% formic acid in acetonitrile. In total, 50 μL of each extracted sample was injected onto the column with a starting flow of 150 μL/min and an organic phase (B) gradient at 0 min, 15% and 0.6 min, 15%; after a flow increase to 300 μL, the gradient of the organic phase continued at 0.7 min, 28%; 4.5 min, 35%; 4.6 min, 35%; 4.8 min, 90%; 5.5 min, 90%; 5.7 min, 15%; and 7.4 min, 15%; and finally, flow was decreased to 150 μL/min with a gradient at 7.5 min, 15%. The autosampler temperature was set to 4 °C. To verify robustness, four different LC columns from two different lots (Lot 040-15-007 S# 001369 and 001375; Lot 023-15-007 S# 001350 and S# 001371) were used during the validation procedure.

In contrast to a previously published method [15], both the change in column and gradient were altered, resulting in an analysis time reduced by 50%. Note that using this former method, quantification of IDeg was not possible due to unfavourable interactions with the stationary phase, most likely the result of the lipidation of IDeg in comparison with other insulins.

The chromatographic system was coupled to a Q Exactive Plus hybrid quadrupole-Orbitrap mass spectrometer (Thermo Scientific, USA) working in positive ion mode with a spray voltage of 4.5 kV and an inlet capillary temperature of 300 °C. The instrument was set to operate in full scan mode acquiring full scan data MS with a resolution setting of 70,000 (at *m*/*z* 200) in a mass range of 1100–1600 Da (AGC target 1e6 and maximum injection time 200 ms).

### Data analysis

**Fig. 1:**
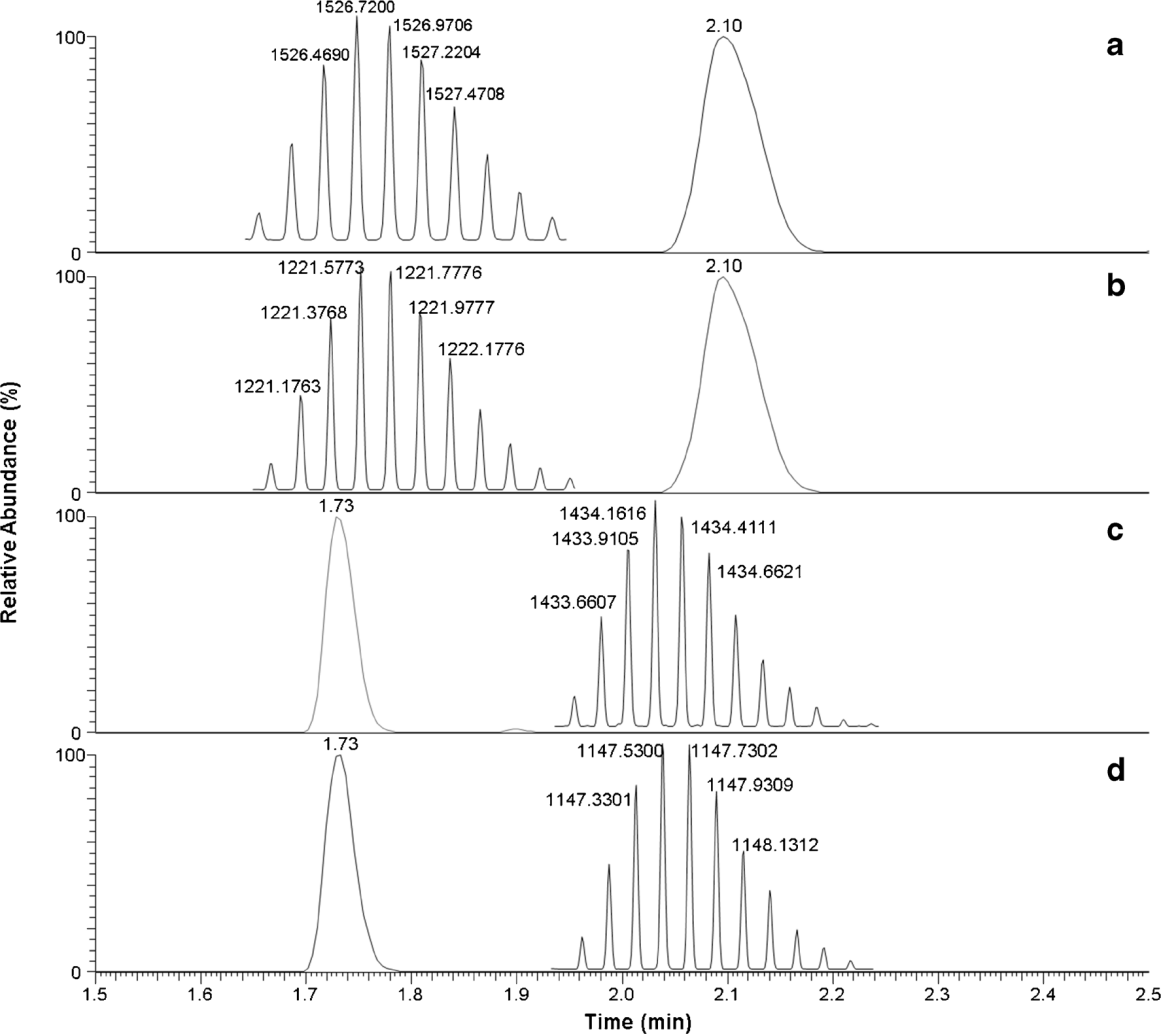
Representative LC-MS data of simultaneous extraction and detection of insulin degludec and bovine insulin from charcoal-treated serum spiked at a concentration of 2000 pM. The mass spectra are averaged across the chromatographic peak. The insets show the isotopic distributions of the corresponding charge state averaged over the chromatographic peak. **a** Insulin degludec 4+ charge state. **b** Insulin degludec 5+ charge state. **c** Bovine insulin 4+ charge state. **d** Bovine insulin 5+ charge state

The most abundant charge states and the most abundant isotopes per each charge state were used for quantification (Fig. 1 and Table 1). The summation of the most abundant isotopes per each of the 4+ and 5+ charge states was combined to provide the total AUC (area under the curve) for IDeg and the bovine insulin used as ISTD. IDeg quantification was based on a calibration curve generated by plotting the concentrations of IDeg versus the ratio of the AUCs of IDeg and bovine insulin. Mass tolerance of all *m*/*z* values was set to 5 ppm. All data were processed using Pinpoint 1.3 and TraceFinder 4.0 software (Thermo Scientific, USA).

**Table 1:** *m/z* values of the ions summed for quantification of insulin degludec and bovine insulin (theoretical values)

Insulin degludec	*m*/*z* values	Bovine insulin	*m*/*z* values
4+	1526.4677	4+	1433.6582
	1526.7182		1433.9088
	1526.9687		1434.1593
	1527.2191		1434.4097
			1434.6601
5+	1221.3756	5+	1147.3285
	1221.5760		1147.5289
	1221.7764		1147.7292
	1221.9767		1147.9295
	1222.1771		1148.1298

### Method validation

The method was validated according to FDA guidelines for bioanalytical method validation [16] for the following parameters: lower limit of quantification (LLOQ), linearity, accuracy, precision, selectivity/specificity, carry-over, recovery and stability.

#### Lower limit of quantification

The LLOQ of the assay was calculated analysing replicates (*n* = 15) of spiked charcoal–treated serum samples at 120 pM on three different days spread over 2 weeks. Replicates (*n* = 2) of the zero calibrators (blank serum spiked with internal reference only) were injected in each same run for comparison.

#### Linearity

Linearity was determined by spiking charcoal-treated serum (blank matrix) with 120, 250, 500, 1000, 2000, 4000, 6000 and 8000 pM of IDeg in duplicate. A blank (no sample, no ISTD) and a zero calibrator (blank plus ISTD) in the same serum matrix were included also in the run. Independent calibration curves were prepared and analysed on three different days across 2 weeks.

#### Accuracy and precision (intra-assay and inter-assay)

The intra-assay accuracy and intra-assay precision of the method were evaluated by preparing replicates (*n* = 15) of QC samples at 120 pM (LLOQ), 360 pM (low), 1500 pM (mid) and 7000 pM (high). In the same run, a duplicate calibration curve containing the same calibrants as for linearity (see above) was used to calculate the QC concentrations. Inter-assay accuracy and inter-assay precision were calculated based on the replicate determination of each concentration level made on three separate days spread across 2 weeks (*n* = 15 at each concentration and on each day).

#### Selectivity/specificity

The selectivity and specificity of the method were tested using six blank samples from different commercial sources. These blank matrices were fortified with the insulin analogues IGlu, IAsp or ILisp to check interference on method performance from potential concomitant medication. Specifically, the pooled disodium EDTA plasma and the pooled serum were spiked with 600 pM of IGlu; the pooled sodium heparin plasma and the dipotassium EDTA plasma were spiked with 600 pM of IAsp and the two non-pooled serums were spiked with 600 pM of ILisp. The presence of interference at the retention times of the analyte (IDeg) and internal standard (bovine insulin) was assessed. The ISTD response in the blank was also tested to not exceed 5% of the average ISTD response of the calibrators and QCs. Additionally, the ability of the assay to accurately and precisely measure IDeg in the presence of IGlu, IAsp and ILisp, each spiked at a concentration of 600 pM, was evaluated. This concentration was selected based on expected peak levels of these rapid analogues in a representative insulin-treated patient without severe insulin resistance. IDeg QC samples in replicates of six were prepared at four concentration levels, 120 pM (LLOQ), 360 pM (low), 1500 pM (mid) and 7000 pM (high) in the presence of other analogues at the indicated concentration. Additionally, QC samples were also prepared in pooled human serum at concentration levels of 360 pM, 1500 pM and 7000 pM (*n* = 14 at each concentration) for the assessment of accuracy and precision in a more complex matrix (ESM Table S2).

#### Carry-over

The carry-over was determined by analysing blank samples (*n* = 2) after injection of the calibrant at the highest concentration (8000 pM) on three different days throughout 2 weeks. In the same batch, replicate analyses (*n* = 5) of analyte at the LLOQ of the assay were included for comparison.

#### Recovery

The reproducibility of the analyte recovery was studied by analysing five replicate QC samples spiked at three concentration levels, 360 pM (low), 1500 pM (mid) and 7000 pM (high). These samples were compared to five replicate blank sample extracts spiked with the same concentrations of the analyte post-extraction and before LC-MS injection.

#### Stability

The stability of the analyte in the post-elution preparation before LC-MS injection was assessed at times 0 h, 24 h and 48 h. For each day, a fresh calibration curve was prepared with each calibration point in duplicate. Four replicates of QC samples at two concentration levels, 360 pM (low) and 7000 pM (high), were tested at each time. Extracted samples were stored in an LC-MS autosampler at 4 °C for the indicated times before analysis.

#### Robustness

Throughout the validation procedure, MSIA D.A.R.T.’S from three different lots and four different LC columns were used. Additionally, the accuracy and precision experiments were performed independently by two different technicians. None of these parameters had any impact on assay performance.

## Results

Our workflow consists of three steps: analytes are extracted from serum or plasma by immunopurification, then separated by liquid chromatography and finally detected by mass spectrometry. Representative LC-MS data is shown in Fig. 1. The internal standard bovine insulin is eluted at the retention time of 1.7 min, IDeg after 2.1 min. Due to their different molecular sum formulas, both compounds exhibit different masses and therefore also compound-specific *m*/*z* values are detected by the mass spectrometer. Due to the highest signal intensity, the *m*/*z* values corresponding to 4+ and 5+ charge states of the analytes were selected for analysis. These *m*/*z* values are listed in Table 1.

We carried out method validation according to the FDA guidelines on bioanalytical method validation (see “Materials and methods” for details). The lower limit of quantification (LLOQ) was established at 120 pM (accuracy 7.8–16% error, precision 2.5–11.3% CV) and linearity was confirmed up to a concentration of 8000 pM (*r*^2^ = 0.99 for three independent runs; 95% of all calibrators (*n* = 70) within ± 15%). Intra-assay accuracy and inter-assay accuracy were maintained over the entire calibration range: all samples were within ± 10% of theoretical values (*n* = 60), thereby clearly outperforming the target criteria (± 15%; Table 2). Similarly, intra-assay precision and inter-assay precision were below 10% CV at all tested concentrations (Table 2).

**Table 2:** Intra- and inter-assay accuracy and precision (*n* = 15 for each concentration)

		QC concentration (pM)			
		120	360	1500	7000
Accuracy (error %)	Intra-assay	− 3.0	0.0	0.0	− 2.0
	Inter-assay	5.0	− 1.0	− 2.0	2.0
Precision (CV %)	Intra-assay	5.7	6.2	3.9	5.7
	Inter-assay	3.8	6.2	5.1	7.7

**Fig. 2:**
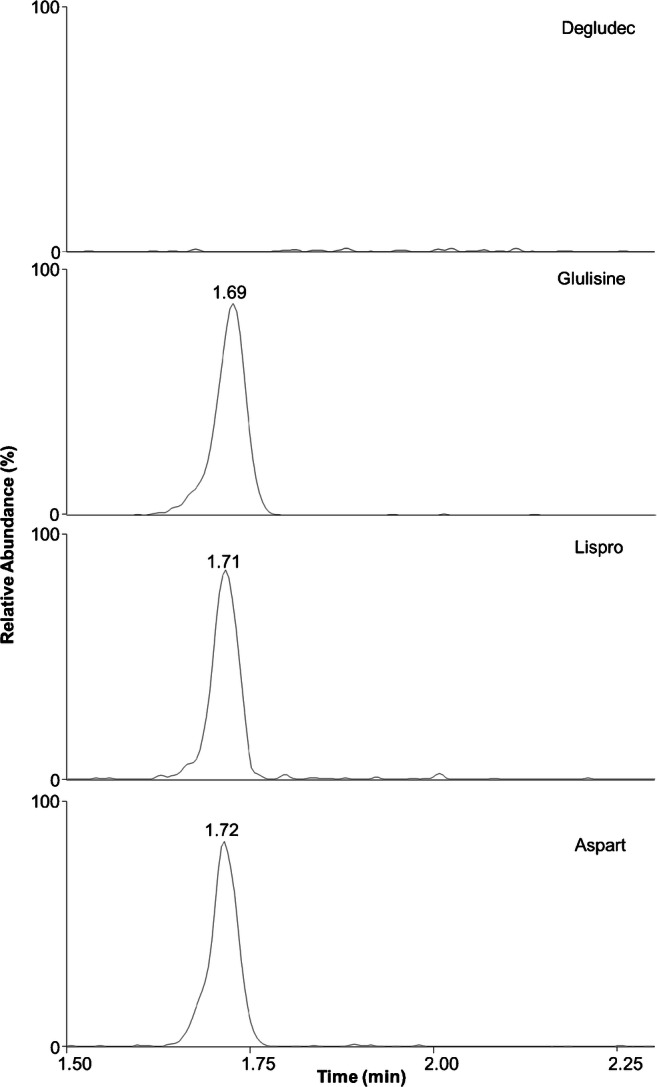
Extracted ion chromatograms (four most abundant isotopes of the 5+ charge state for each compound) of human serum spiked with the insulin analogues glulisine, lispro and aspart at 600 pM each. The extracted ion chromatogram of degludec is the sum of the measurements of the other insulin analogues. The absence of a peak in the top panel demonstrates that none of the other analogues causes interference with the degludec measurement. Besides having different mass and therefore *m/z* values, the other insulin analogues also display clearly different retention times compared with degludec

**Fig. 3:**
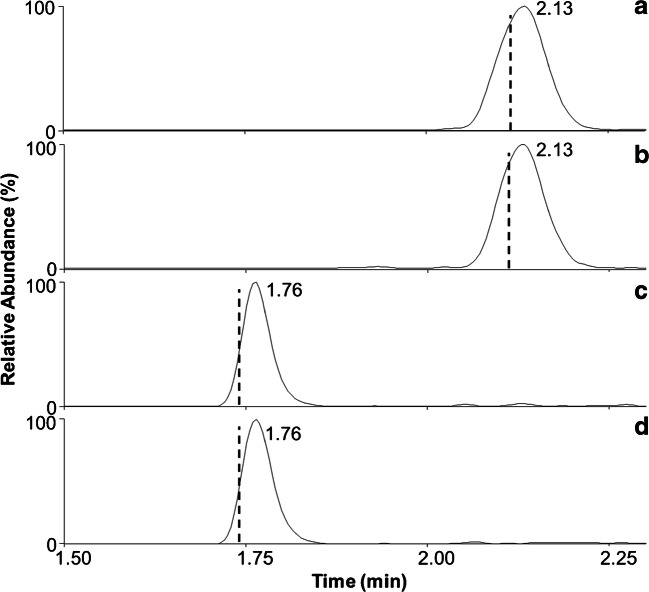
Extracted ion chromatograms of degludec (**a** and **b** for the 5+ and 4+ charge states, respectively) and bovine insulin (**c** and **d** for the 5+ and 4+ charge states, respectively) extracted from a patient sample. Dotted lines indicate the peak maximum of the analytes in stripped plasma (cf. Fig. 1). Even in complex clinical samples, no interference for either compound is detected

It is crucial for clinical assays to be free of interference, as characterized by the selectivity and specificity of the method. Selectivity was confirmed by the absence of signals at the retention times of IDeg and the internal standard in six independent serum and plasma samples. Specificity was investigated in the same samples fortified with commonly used rapid-insulin analogues (IGlu, IAsp and ILisp) at a clinically relevant concentration of 600 pM. The first step of our workflow is unspecific due to the antibody used for immunopurification: it extracts IDeg as well as the other insulin analogues, IGlu, IAsp and ILisp. Specificity is then introduced by the following LC-MS step: IGlu, IAsp and ILisp display clearly different retention times compared with IDeg and all analytes have different mass values, and therefore different *m*/*z* values are detected by the mass spectrometer; consequently, the measurement of IDeg is not interfered by these compounds (Fig. 2). Lack of interference was also confirmed in clinical samples (Fig. 3).

Moreover, we evaluated whether the presence of IGlu, IAsp or ILisp at 600 pM has an effect on accuracy and precision for the measurement of IDeg (Table 3). Except for the lowest tested IDeg concentration (120 pM) for which accuracy was in the range of 25% error, accuracy and precision were maintained at the other IDeg concentrations (360, 1500 and 7000 pM; see Table 3). Note that low IDeg level of 120 pM reflects an unlikely clinical scenario given that reported steady-state concentrations of IDeg are consistently between 3000 and 8000 pM at doses between 0.4 and 0.8 U/kg body weight independent of age groups [5]. To verify robustness and lack of matrix effect, a set of QC samples was prepared in pooled human serum. The results presented in Table S2 (see ESM) clearly show that accuracy and precision are still maintained in this real-world matrix and that parallel detection of endogenous insulin is feasible (ESM Fig. S1).

**Table 3:** Accuracy and precision of degludec in the presence of additional insulin analogues (*n* = 6 at each concentration)

QC (pM)	600 pM glulisine	600 pM aspart	600 pM lispro
Accuracy (error %)			
120	28.6*	26.6	25.7
360	12.4	− 5.1	− 6.8
1500	− 14.0*	− 15.3	− 16.2
7000	− 0.7	− 5.0	− 6.0
Precision (CV %)			
120	11.8*	6.9	6.0
360	8.2	5.3	6.0
1500	10.2*	4.0	7.2
7000	8.4	5.7	4.4

*n = 5

Finally, carry-over (4% of the LLOQ signal), recovery (89.7–97.2%) and stability (accuracy 9.1% error, precision < 8.2% CV) (Table 4) were also confirmed to be within the limits defined by the FDA guidelines.

**Table 4:** Degludec stability after elution and before injection into the instrument

Autosampler storage time	0 h		24 h		48 h	
QC (pM)	360	7000	360	7000	360	7000
Accuracy (error %)	− 5.5	0.3	− 9.1	− 1.1	2.7	− 2.1
Precision (CV %)	5.4	0.5	6.6	4.1	4.6	8.2

**Fig. 4:**
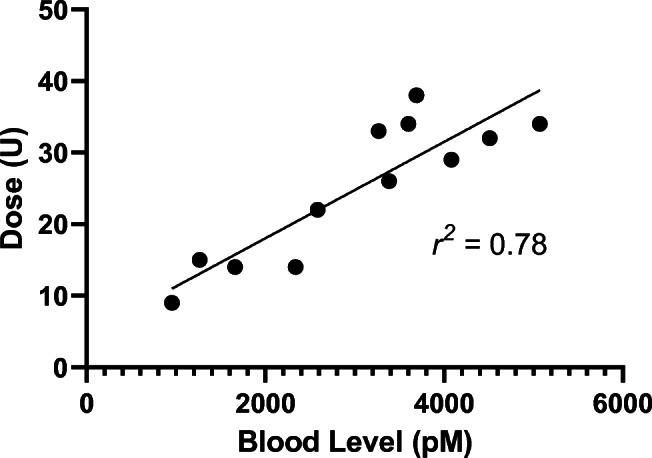
Quantification of insulin degludec (IDeg) in clinical samples. The measured IDeg concentration correlates with the administered dose

Applicability of the method was tested in clinical samples of patients with type 1 diabetes using IDeg at an average dose of 0.3 U/kg body weight. Mean ± SD concentrations of 3035 ± 1238 pM (*n* = 12) were found and we observed a positive correlation (*r*^2^ = 0.78, *p* < 0.001) between the administered IDeg dose and measured IDeg blood levels (Fig. 4) in line with previous reports [5].

## Discussion

In the present work, we developed and validated an automated, high-throughput method for quantification of IDeg in human blood samples by combining immunopurification with liquid chromatography high-resolution mass spectrometry. The novel method satisfyingly met the FDA criteria by showing adequate accuracy, specificity and linearity covering the range of expected therapeutic IDeg concentrations in human samples.

Compared with other LC-MS-based methods for insulin quantification [12], the present approach uses a small sample volume of 250 μL and includes a simple and automated sample preparation, both contributing to its high usability. Thanks to its ease of use and rapid run time (7.5 min per sample or 12 h to run a 96-well plate), the method is highly suitable for studies with a large number of blood samples such as pharmacokinetic studies. The need for reliable and standardized insulin analytics to support the performance of pharmacokinetic studies becomes particularly evident in view of the recent and upcoming expiration of patent protection for a number of insulin preparations. This will open up the insulin market worldwide to manufacturers of insulin generics or biosimilars [17–19]. Demonstration of pharmacokinetic equivalence will be a central part of the approval pathway. Additionally, a number of novel devices targeting minimally invasive insulin delivery at high precision will require in vivo validation studies with accurate insulin quantification [20], especially in view of the new Medical Device Regulation coming into force in May 2021 in Europe [21].

Despite the many clinical benefits of IDeg and hence increasing popularity, the underlying complex biochemical structure poses challenges to its measurement. Commercial immunoassays for human insulin show limited use for IDeg and the presence of a fatty acid modifies its chromatographic behaviour compared with other insulin analogues, thereby affecting the performance of LC-MS-based insulin assays [14]. In the present work, chromatographic conditions were therefore carefully optimized to circumvent this issue while still keeping analysis time short.

Due to the fact that IDeg is often co-administered with rapid-acting insulin analogues (IGlu, IAsp and ILisp), we ensured that the assay also shows adequate performance in the presence of these agents at clinically relevant therapeutic concentrations. Besides having different mass and *m*/*z* values, these other insulin analogues also display clearly different retention times to IDeg. The capacity of multiplexing different types of insulin within a single sample measurement will pave the way towards more complex investigations simultaneously exploring pharmacokinetics of IDeg and other insulin analogues in patients using combination regimens.

We acknowledge that our work is not without limitations. First, IDeg and the other insulin analogues have been assessed in vitro using the parent drug. However, reference standards are not widely available, nor are isotopically labelled internal reference standards. As such, it would be useful for providers of reference materials to consider the production of insulin analogues and their degradation products for inclusion into quantitative workflows such as ours in the future. Second, a comprehensive assessment of the assay’s multiplex capacity and performance warrants further experiments, particularly the inclusion of the full concentration range of rapid-acting insulin analogues and human insulin.

In conclusion, the increasing number of people requiring insulin worldwide, the growing diversity of insulin formulations on the market and multiple novel insulin delivery systems highlight the need for reliable, specific and high-throughput mass spectrometric approaches to quantify insulin analogues in human blood samples. The advantageous pharmacokinetic properties of IDeg have turned it into an attractive drug for clinical use. Still, a bioanalytical method that allows accurate and robust quantification in human blood samples was lacking up to now. Within this study, we introduced a novel, automated, high-throughput antibody-based affinity capture LC-MS method to quantify IDeg in clinical specimen lacking up to now. The method fulfils all FDA validation criteria and, combined with its ease of use, offers new avenues for academic and industrial research in the field of diabetes.

## Electronic supplementary material

ESM 1 (PDF 165 kb)

## Data Availability

Data are available upon reasonable request.
