# Supplementary material for: Rapid quantification of insulin degludec by immunopurification combined with liquid chromatography high-resolution mass spectrometry
Source: Anal Bioanal Chem. 2020 Oct 2;412(30):8351–9. doi: 10.1007/s00216-020-02971-4 (PMC7680744; doi:10.1007/s00216-020-02971-4)
Supplement: Supplementary file 1 — (PDF 165 kb) [file 216_2020_2971_MOESM1_ESM.pdf]

Analytical and Bioanalytical Chemistry

Electronic Supplementary Material

Rapid quantification of insulin degludec by immunopurification combined with liquid chromatography high-resolution mass spectrometry

Gemma Reverter-Branchat, Michael Groessl, Christos T Nakas, Jean-Christophe Prost, Kwasi Antwi, Eric E. Niederkofler, Lia Bally

Table S1 Analyte capture and elution settings for the Degludec MSIA Versette protocol

Procedure	Plate content	Aspirate/dispensed volume (μL)	Number of cycles
**Step 1: MSIA D.A.R.T.'S pre-rinse**	10 mM PBS pH=7.4	150	20
**Step 2: Degludec capture**	Calibrants, quality controls and serum samples	250	100
**Step 3: MSIA D.A.R.T.'S rinse**	10 mM PBS pH=7.4	150	20
**Step 4: MSIA D.A.R.T.'S rinse**	10 mM PBS pH=7.4	150	20
**Step 5: MSIA D.A.R.T.'S rinse**	Water	150	20
**Step 6: MSIA D.A.R.T.'S rinse**	Water	150	20
**Step 7: Degludec elution**	0.4% TFA / 33% ACN / 450 μg/mL Leucine Enkephalin / 0.025 mg/L BSA	50	100
Total time: 1h 25min			

Table S2 Assessment of accuracy and precision IDeg spiked into pooled human serum samples (BioclamationIVT, Lot # BRH841923). N = 14 at each concentration level

Theoretical Concentration	Average Experimental Concentration	Accuracy (Error %)	Precision (CV %)
360 pM	313 pM	-13%	12%
1500 pM	1353 pM	-10%	10%
7000 pM	7247 pM	4%	10%

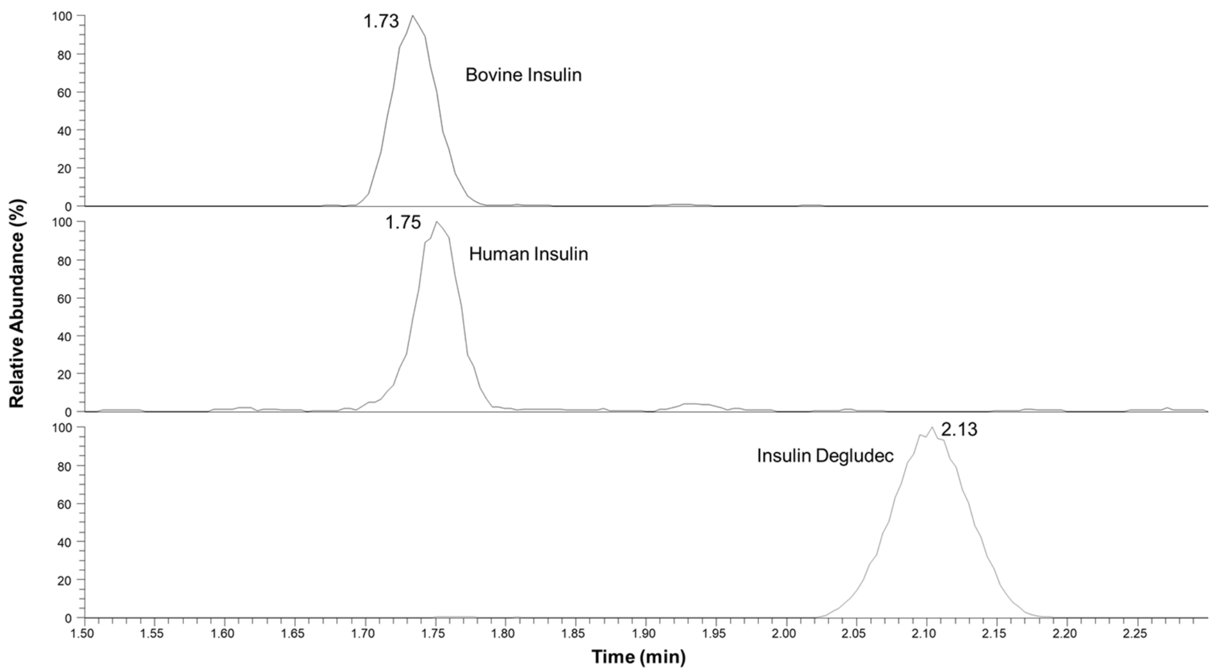

Fig. S1 Parallel detection of human insulin and Insulin Degludec in a pooled human serum sample (BioclamationIVT, Lot # BRH841923). Insulin Degludec was added at a concentration of 1500 pM. The summed abundance of the five most abundant isotopes of the 5+ charge state of each compound is displayed
